# The Roles of CD38 and CD157 in the Solid Tumor Microenvironment and Cancer Immunotherapy

**DOI:** 10.3390/cells9010026

**Published:** 2019-12-20

**Authors:** Yu Jun Wo, Adelia Shin Ping Gan, Xinru Lim, Isabel Shu Ying Tay, Sherlly Lim, Jeffrey Chun Tatt Lim, Joe Poh Sheng Yeong

**Affiliations:** 1 Yong Loo Lin School of Medicine, National University of Singapore, Singapore 117597, Singapore; yujunwo@gmail.com; 2 Lee Kong Chian School of Medicine, Nanyang Technological University, Singapore 636921, Singapore; SGAN012@e.ntu.edu.sg; 3 Institute of Molecular and Cell Biology (IMCB), Agency of Science, Technology and Research (A*STAR), Singapore 138673, Singapore; limxr@imcb.a-star.edu.sg (X.L.); sherllyl@imcb.a-star.edu.sg (S.L.); limct1@imcb.a-star.edu.sg (J.C.T.L.); 4 School of Applied Science, Temasek Polytechnic, Singapore 529765, Singapore; isabeltay123@gmail.com; 5 Division of Pathology, Singapore General Hospital, Singapore 169856, Singapore

**Keywords:** CD38, CD157, TME (Tumor Microenvironment), Cancer Immunotherapy, Immunotherapy Targets

## Abstract

The tumor microenvironment (TME) consists of extracellular matrix proteins, immune cells, vascular cells, lymphatics and fibroblasts. Under normal physiological conditions, tissue homeostasis protects against tumor development. However, under pathological conditions, interplay between the tumor and its microenvironment can promote tumor initiation, growth and metastasis. Immune cells within the TME have an important role in the formation, growth and metastasis of tumors, and in the responsiveness of these tumors to immunotherapy. Recent breakthroughs in the field of cancer immunotherapy have further highlighted the potential of targeting TME elements, including these immune cells, to improve the efficacy of cancer prognostics and immunotherapy. CD38 and CD157 are glycoproteins that contribute to the tumorigenic properties of the TME. For example, in the hypoxic TME, the enzymatic functions of CD38 result in an immunosuppressive environment. This leads to increased immune resistance in tumor cells and allows faster growth and proliferation rates. CD157 may also aid the production of an immunosuppressive TME, and confers increased malignancy to tumor cells through the promotion of tumor invasion and metastasis. An improved understanding of CD38 and CD157 in the TME, and how these glycoproteins affect cancer progression, will be useful to develop both cancer prognosis and treatment methods. This review aims to discuss the roles of CD38 and CD157 in the TME and cancer immunotherapy of a range of solid tumor types.

## 1. Background

Cancer immunotherapy has been advancing exponentially [1,2]. Identification of targets in the biological pathways of tumor cells successfully led to development of monoclonal antibody and tyrosine kinase inhibitor drugs, now actively used in cancer treatment. This has provided patients with additional treatment options and in certain instances, improved their cancer prognosis. However, as the number of patients benefitting from immunotherapy is suboptimal, many studies have focused on discovering novel biomarkers to reliably identify potential responders [3,4] Classification of the immune infiltrates within the tumor microenvironment (TME) would enable more accurate prediction of cancer prognosis [5,6,7,8]. In cancer, immune cells present within the TME may either promote or inhibit tumor growth and development [5,9]. Surface glycoproteins expressed by immune infiltrates can be used as biomarkers for classification of the immune cells. These glycoproteins also influence the pro- or anti-tumor activity of immune cells. Thus, the presence and functions of glycoproteins on the surface of tumor immune infiltrates are currently subjected to intense study.

CD38 and CD157 are two such glycoproteins of particular interest in the field of immunotherapy. They are coded by contiguous gene sequences found on human chromosome 4, and are thought to originate from gene duplication. These gene sequences share similarities in terms of length and the organization of introns and exons, and the resultant proteins share similar functions [10]. CD38 and CD157 function as both receptors and ectoenzymes, and belong to the same family of nicotinamide adenine dinucleotide (NAD+) converting enzymes. 

CD38 is involved in lymphocyte activation, proliferation and adhesion. Initially thought to be expressed only by thymic lymphocytes, it has since been found to be ubiquitously expressed by immune cells, including B lymphocytes, natural killer cells and monocytes; and its expression varies across both lymphoid and non-lymphoid tissues [10,11]. In contrast, CD157 is mainly expressed by cells derived from the myeloid lineage, and in particular by neutrophils and monocytes. CD157 is also expressed by a wide range of non-lymphoid tissues, including vascular endothelium, kidney collecting tubules and Paneth cells in the stomach [12]. 

Both CD38 and CD157 have been used as therapeutic targets in clinical trials to treat solid tumors [12,13,14,15]. This review aims to give an overview of their roles of in the TME, which might provide insights for therapeutic strategies across various cancers. Information on the roles of CD38 and CD157 in different cancers is consolidated from relevant data and evidence available in existing literature.

### 1.1. The Role of CD38 in the TME

**Figure 1:**
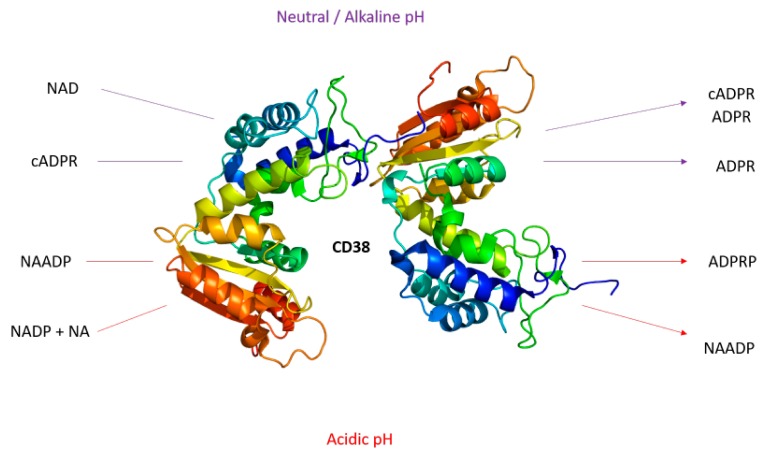
Pictorial summary of reactions catalyzed by CD38 at different pH levels.

The first indication that CD38 is an enzyme came from the discovery of similarities in amino acid sequences between CD38 and ADP-ribosyl cyclase from the genus *Aplysia*. In *Aplysia*, ADP-ribosyl cyclase catalyzes the cyclization of NAD+, a linear molecule, to form cyclic ADP-ribose (cADPR) [11]. Similar to ADP-ribosyl cyclase, CD38 catalyzes the conversion of NAD+ to cADPR. While the majority of the NAD+ catalyzed by CD38 is converted to ADPR, a minority is cyclized to form cADPR [11]. However, CD38 is a multifactorial ectoenzyme, and acts on NADP+ in addition to NAD+. At an acidic pH, CD38 catalyzes the synthesis of nicotinic acid adenine dinucleotide phosphate (NAADP) from NADP+ [10,11]. NAADP can also be used as a substrate by CD38, which converts it into ADP-ribose 2′-phosphate (ADPRP) (Figure 1).

cADPR and NAADP are secondary messengers involved in calcium regulation in plants, protists and animals, including humans. Calcium regulation results in the activation of signaling pathways that control a wide range of physiological functions, including lymphocyte proliferation, insulin release by the pancreas, cardiac muscle contraction, neutrophil chemotaxis and T cell activation. These pathways were impaired in CD38-knockout mice, evidenced by reduced immune responses and cardiac hypertrophy [11].

In solid tumors, hypoxic regions form in the TME during tumor growth and development. This is the result of poor blood supply and increased oxygen consumption, which ensue from hyperplasia [16,17]. In the hypoxic TME, NAD+ is produced by the salvage pathway and is metabolized by ectoenzymes to form adenosine, a nucleoside that regulates the immune response. In hypoxic conditions, adenosine suppresses the anti-tumor immune response by recruiting myeloid-derived suppressor cells and T regulatory cells, which inhibits the activity of T effector cells and thus favors tumor progression [18,19]. CD38 is one of the three ectoenzymes involved in the production of adenosine through the CD38/CD203a/CD73 adenosinergic pathway. A high CD38 expression in the TME is thus likely to confer a poor prognosis. Furthermore, CD38 has been found to be expressed by immune infiltrates, such as macrophages and newly activated cytotoxic T cells, in the TME of both primary and metastatic tumors [6,20].

In the first step of the adenosinergic pathway, CD38 hydrolyses NAD+ to form ADPR either directly or through the intermediate cADPR. ADPR is subsequently hydrolyzed by CD203a to form AMP. CD203a can also use NAD+ directly as a substrate to form AMP. AMP is then phosphorylated to adenosine by CD37 [6,21,22]. Extracellular adenosine then binds to adenosine receptors on immune cells, including T cells, natural killer cells, neutrophils, macrophages and dendritic cells, preventing their activation [6,18]. This inhibits both the innate and the adaptive immune response, and results in an immunosuppressive TME. Adenosine further suppresses the anti-tumor immune response under hypoxic conditions by recruiting myeloid-derived suppressor cells and T regulatory cells, further inhibiting the activity of T effector cells and favoring tumor progression [18,19]. Previous studies have shown that immunosuppression in the TME can be reduced by inhibiting either the enzymes or receptors in the adenosinergic pathway. The introduction of oxygen to reverse hypoxia can also significantly slow tumor growth. These promising findings suggest that the CD38/CD203a/CD73 adenosinergic pathway represents an effective target for cancer immunotherapy [6,23].

However, in addition to catalyzing the production of adenosine, CD38 may also promote tumor progression by inducing other tumor-supporting processes in the TME. NAADP, which is produced by CD38, has been found to be heavily involved in vascular endothelial growth factor (VEGF)-induced angiogenesis [24]. VEGF is the main angiogenic growth factor and binds to its receptors, VEGFR1 and VEGFR2, to stimulate angiogenesis, and is involved in the vascularization of solid tumors. NAADP is linked to VEGFR2 activation through its involvement in calcium signaling [25,26]. Binding of VEGF to VEGFR2 stimulates the release of Ca^2+^, and NAADP contributes to this pathway by stimulating additional Ca^2+^ release from acidic organelles such as lysosomes and endosomes. The resultant Ca^2+^ influx acts as a second messenger to promote angiogenesis, which in turn promotes tumor growth and metastasis [27]. The inhibition of angiogenesis is a potential treatment modality for cancer, and this may be achieved through the inhibition of NAADP production by CD38.

CD38 also functions as a cell surface receptor, and serves an important role in the activation and proliferation of immune cells [10,24]. Anti-CD38 antibodies have been shown to prevent the adhesion of CD4+ T cells to endothelial cells, suggesting that CD38 is involved in leukocyte adhesion and migration [28]. In addition, CD38 promotes the activation, proliferation and survival of lymphocytes, as well as the release of pro-inflammatory and regulatory cytokines from dendritic cells and monocytes [10,29]. CD38 is also expressed by monocytes, where it serves as a coreceptor in the MHC Class II-mediated activation of T cells by superantigens [30]. Co-expression of CD38 and human leukocyte antigen-DR isotype (HLA-DR) indicates the presence of recently activated effector CD8+ T cells [20,31,32]. The number of HLA-DR and CD38-coexpressing CD8+ T cells can be up to five times higher within the tumor compared to the peripheral blood of cancer patients [32]. CD38 can hence serve as a biomarker for monitoring immune response against tumors. With the development of multiplexing techniques for immunohistochemistry and immunofluorescence, it is now possible to identify up to 30 discrete antigens within a tissue sample [33,34]. Greater knowledge of the roles and presence of biomarkers like CD38 in relation to cancer progress could capitalize on the efficiency offered by multiplexing techniques, and ultimately enhance cancer prognosis and provide timely treatment. It is clear that the dual enzymatic and receptorial functions of CD38 mean that this glycoprotein affects the immune response in a number of ways, and these must be fully characterized to improve the accuracy of cancer prognosis and efficacy of treatment.

### 1.2. The Role of CD157 in the TME

Similar to CD38, CD157 is a β-NAD+ metabolizing ectoenzyme. CD157 has readily detectable NAD+ glycohydrolase activity, which catalyzes the production of ADPR from NAD+. Its role in ADPR production suggests the potential involvement of CD157 in the CD38/CD157/CD203a/CD73 adenosinergic pathway [35], which as aforementioned, results in increased adenosine levels and an immunosuppressive TME that favors tumor progression [18,19]. Both CD38 and CD157 have ADP-ribosyl cyclase activity and can convert NAD+ into cADPR, although the cyclase activity of CD157 is hundred times less efficient than that of CD38 [10,36]. However, CD157’s hydrolase and cyclase activities are significantly increased by an acidic environment, as well as by the presence of zinc and magnesium ions [10,12,36]. cADPR is a potent mediator of calcium release into the cytosol, and the ability of CD157 to synthesize cADPR implies that, like CD38, it is involved in calcium homeostasis. Furthermore, CD157 may be involved in a broad range of physiological functions, including lymphocyte proliferation, neutrophil chemotaxis, T cell activation and intestinal smooth muscle contraction [37].

CD157 also serves as a receptor and, like CD38, is capable of transducing intracellular signals. This function is mediated by its heparin-binding domains, which bind with high affinity to certain elements in the extracellular matrix [12]. Furthermore, CD157 has an essential role in the immune response and is expressed by bone marrow stromal cells, where it promotes pre-B cell growth [10,38]. It is also constitutively expressed by mononuclear peripheral blood cells from the myeloid lineage [36]. However, this expression is only observed on mature cells and not on precursors, suggesting that CD157 may be involved in myeloid differentiation [39].

CD157 also has a role in leukocyte trafficking; it mediates myeloid cell adhesion, migration and diapedesis during an inflammatory response [10,37]. Its involvement in cell adhesion is in line with its expression on the cell membranes of leukocytes, mesothelial cells and vascular endothelial cells [12,37,40]. Experiments investigating the role of CD157 in neutrophil migration and adhesion incubated neutrophils with monoclonal antibodies (mAbs) against CD157, and found that the migration of these neutrophils was significantly inhibited. Similarly, neutrophil adhesion to extracellular matrix proteins was also significantly decreased by CD157 ligation, suggesting that CD157 ligation decreases both immune cell adhesion and trafficking [41]. Furthermore, CD157 is also expressed by cancer cells, and it may mediate tumor cell invasion and metastasis in the same manner as it does leukocyte trafficking [37]. This may explain why CD157 expression on tumor cells is an indication of poor prognosis in ovarian cancer and malignant pleural mesothelioma [12].

## 2. CD38 and CD157 as Targets for Cancer Immunotherapy

Cancer is a notoriously heterogeneous disease, with the characteristics of tumor cells differing both between different types of cancer and within each tumor type. This makes treatment a challenge, and while drugs that target tumor cells directly have been found to elicit therapeutic effects in certain cancers, these drugs typically have restricted applications and efficacy. Furthermore, tumors tend to have a TME that sustains and promotes tumor proliferation, even in the face of targeted treatment [42]. This necessitates the development of alternative drugs that target cells within the TME instead, such as certain classes of immune infiltrates.

Cancer cells are known to suppress the anti-tumor immune response using certain regulatory pathways. The two most well-understood pathways involve programmed cell death protein 1 (PD-1) and cytotoxic T-lymphocyte protein 4 (CTLA4). PD-1 is present on the surface of activated T cells in the TME, while its ligand, programmed death ligand 1 (PD-L1), is expressed in large quantities on the surface of tumor cells. When PD-L1 binds to PD-1 on activated T cells, they are deactivated, inhibiting the anti-tumor immune response [8]. CTLA-4, which is expressed by activated T cells, is also involved in immunosuppression through competition for substrates with its homolog, CD28. B7, which is expressed by antigen presenting cells, must bind to CD28 expressed by T cells to activate them. However, CTLA-4 has a greater affinity for B7 than CD28 and is able to outcompete it, transmitting inhibitory signals and preventing T cell activation [8,43]. This also serves to suppress anti-tumor immune responses.

Blocking these regulatory pathways have been found to boost anti-tumor responses in mice, proving their relevance to the field of cancer immunotherapy [8]. However, these two pathways alone do not account for all the immunosuppression occurring in the TME, with other factors and pathways that mediate the same effect also being present. Furthermore, only a small percentage of cancer patients benefit from immunotherapy in the long run, due to the development of resistance after an extended period of treatment [44]. This emphasizes the importance of understanding the roles of factors such as CD38 and CD157, which are involved in immunosuppression, to successfully predict and alter the immune response to various types of tumor. Due to their diverse functions in the TME, CD38 and CD157 may be particularly useful as both prognostic tools and therapeutic targets. Table 1 and Table 2 show a summary of the known functions of CD38 and CD157 in a number of types of cancer, and the next sections of this review will discuss these in-depth.

### 2.1. CD38 in the Solid Tumor Microenvironment

#### 2.1.1. CD38 in Hepatocellular Carcinoma

Hepatocellular carcinoma (HCC), the most common form of primary liver cancer, is the third leading cause of cancer-associated mortality worldwide. At present, liver resection and transplantation represent the most established treatment methods for early stage HCC, while advanced-stage HCC is treated by chemotherapy with the kinase inhibitor Sorafenib [58]. However, HCC still has a relatively low five-year survival rate and high incidence of recurrence [30]. Cancer immunotherapy is now being explored as an alternative form of treatment, and studies show promising results. A study with nivolumab, for instance, showed a 74% 9-month overall survival rate among subjects with untreated advanced HCC [59]. There remains a need to improve predictions of patient outcomes and the outcomes themselves, to increase the efficiency of anti-PD-1 treatment.

**Figure 2:**
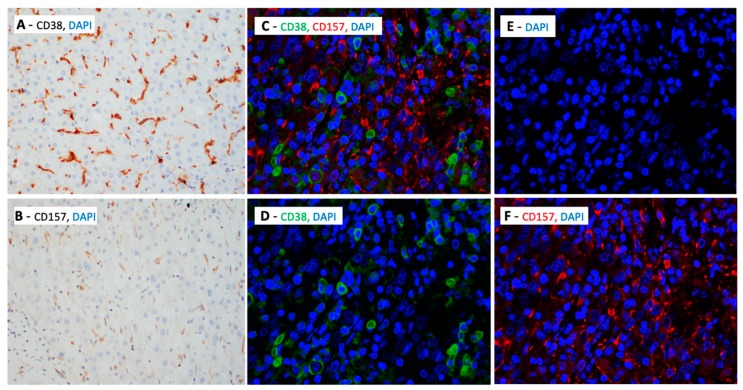
Multiplex immunohistochemistry/immunofluorescence of hepatocellular carcinoma biopsies, showing the expression of (**A**) CD38 and DAPI, (**B**) CD157 and DAPI, (**C**) CD38, CD157 and DAPI, (**D**) CD38 and DAPI, (**E**) DAPI alone and (**F**) CD157 and DAPI (magnification: 400×).

In HCC, CD38+ is found to be expressed both on immune infiltrates within the TME as well as on the HCC tumor cells (Figure 2). Biopsies show unique aggregations of CD38+ tumor infiltrating lymphocytes (TILs) in HCC, and the presence of larger numbers of CD38+ TILs is associated with improved effectiveness of immunotherapy in HCC [6,7]. In one particular study, the use of the PD-1-specific mAb, nivolumab, led to a decrease in tumor size in one-fifth of patients with advanced HCC. Nivolumab works by significantly reducing the numbers of unresponsive T cells, and increasing the numbers of activated T cells expressing CD38 [7]. These activated CD38+ TILs produce cytotoxic compounds and inflammatory cytokines to attack the tumor. These cytokines include interferon gamma (IFN-γ), which serves a critical role in tumor control, upregulating the immune response and exerting pro-inflammatory activity on immune infiltrates and tumor cells [45,46]. The induction of inflammation in the TME promotes an anti-tumor immune response [7]. A previous study demonstrated that higher levels of IFN-γ-producing T cells in the TME of HCC patients is correlated with improved survival following sorafenib treatment [60]. Thus, a correlation can be established between an increased density of CD38+ TILs and improved prognosis in patients with HCC [61]. The high degree of variability in the number and activity of these CD38+ TILs between patients could explain the inter-individual variance in anti-tumor responses following immunotherapy [7]. This suggests that the presence and density of CD38+ immune infiltrates could be used to predict the effectiveness of anti-PD-1 immunotherapy in patients with HCC.

However, previous studies have established that HCC resistance to PD-1/PD-L1 antibodies could be caused by CD38 upregulation on tumor cells [6]. Upregulation of CD38 typically occurs after a period of anti PD-1/PD-L1 treatment. Immunosuppression brought about by the adenosinergic activity of CD38 leads to suppressed cytotoxic T cell function, limiting the therapeutic benefit conferred by anti-PD-1 immunotherapy [6,44]. This suggests that CD38 may be a potential, additional immunotherapeutic target in HCC that can be used alongside PD-1/PD-L1 immunotherapy.

CD38 has also been discovered to be expressed on macrophages, where it serves as a coreceptor in the MHC Class II-mediated activation of T cells [30,62]. The presence of macrophages expressing both CD38 and CD68 in the HCC TME is correlated with improved prognosis after surgical resection, but CD68+ macrophage density is associated with poor prognosis [30]. This may indicate the presence of various macrophage subsets with different functions, where the CD38+ macrophage subset may serve to promote inflammation and exert anti-tumor effects. CD38 is highly expressed by M1 macrophages, which produce large amounts of cytokines such as IL-6 and TNFα. These cytokines induce inflammation and thus contribute to the immune response against the tumor. A previous study observed that the absence of either of these two cytokines led to accelerated HCC development [47]. However, unlike M1 macrophages, M0 and M2 macrophages make no appreciable contribution to the levels of cytokines in the TME [30]. These findings indicate that macrophages expressing CD38 could correspond to the M1 phenotype, and the presence of CD38+ macrophages in the TME may thus be associated with improved prognosis in patients with HCC.

#### 2.1.2. CD38 in Non-Small Cell Lung Cancer

Lung cancer is the most common type of cancer, and the leading cause of cancer-associated mortality across the world [63,64,65]. Non-small cell lung cancer (NSCLC) accounts for 85% of all newly-diagnosed lung cancer cases, but the median survival time for patients with advanced NSCLC is about one year [65]. For these patients, platinum-based chemotherapy is the standard first line of treatment, followed by cytotoxic chemotherapy, but immunotherapy is emerging as a promising method of treatment. The development of agents targeting the CTLA-4 and PD-1 pathways has provided a new lung cancer treatment modality, and a new outlook for these patients.

Considerable progress has been made in the development of anti-PD-1/PD-L1 therapy, and other forms of immunotherapy, which involve checkpoint inhibition. However, the efficacy of these methods is hindered by high rates of resistance that develop following treatment, potentially due to the upregulation of CD38 expression on NSCLC tumor cells. A previous study demonstrated this using Lewis lung carcinoma (LLC) models of NSCLC in mice [44]. In this study, there was initial suppression of tumor growth in models undergoing anti-PD-L1 or anti-PD-1 therapy. However, complete resistance developed by the 5th week of treatment, and by this point the treatment group showed no significant difference in terms of tumor growth compared with the control groups. Genetic and proteolytic analyses were performed, and CD38 was the only markedly upregulated gene or protein identified following anti-PD-L1 therapy. Quantitative polymerase chain reaction and fluorescence-activated cell sorting further confirmed that tumor cells that developed resistance expressed elevated levels of CD38 [44].

Interferon β and all-trans retinoic acid are important mediators of CD38 upregulation in NSCLC cells, enabling tumors to acquire resistance to anti-PD-L1 and anti-PD-1 therapy over a period of time [44]. CD38 upregulation suppresses the activity of cytotoxic T cells via the adenosinergic pathway, reducing their proliferation, cytokine production, and killing capacity. A strong correlation has been found between the presence of CD38 and inflammation in the TME, and anti-PD-1 treatment efficacy is improved when it is used concurrently with antibodies targeting CD38 [44]. The resistance to treatment conferred by CD38 shows the need for further research and trials investigating the therapeutic efficacy of combining anti-CD38 therapy with existing anti-PD-1/PD-L1 treatments.

Previous studies have also shown that CD38 expression on T cells in the NSCLC TME correspond to early effector cells (EECs), which are T cells that are recently activated and non-exhausted. These EECs have also been found to transiently express inhibitor receptors after activation [31]. CD38 may therefore represent a useful biomarker to help monitor the immune response against NSCLC. This function was demonstrated in another previous study, where immunotherapy targeting PD-1 was shown to induce the proliferation of CD38+ CD8 T cells in NSCLC. These CD38+ cells correspond to the effector phenotype. The results revealed that early CD8 T cell proliferation is associated with a positive clinical response to anti-PD-1 immunotherapy; thus, CD38 may be able to predict immunotherapy outcome in NSCLC [66].

#### 2.1.3. CD38 in Melanoma

Malignant melanoma is the deadliest form of skin cancer, and develops from pigment-producing cells in the skin called melanocytes. Although it only comprises 2% of skin cancer cases, it is responsible for 80% of skin cancer-associated mortality [24,67,68]. This deadly form of skin cancer has become increasingly prevalent among fair-skinned populations over the past 5 decades, and treatment modalities and survival rates depend on the stage of the cancer [69]. In its early stages, melanoma can be cured by local excision. However, for unresectable stage III and IV melanoma, conventional treatment methods do not yield a significant increase in survival rates [24]. However, the recent approval of immunotherapeutic agents such as anti-PD-1 and anti-CTLA-4 has achieved unprecedented success in patients with advanced melanoma. A previous study has reported a 3-year survival rate of 63% in patients with advanced melanoma receiving concurrent nivolumab and ipilimumab treatment [70,71]. However, not all immunotherapeutic approaches have been successful and, as in other types of cancer, the efficacy of immunotherapy is still restricted by severe side effects, the development of resistance and a limited response rate [68].

The melanoma TME contributes significantly to tumor progression, and may thus provide a source of effective targets for immunotherapy [18,24,68,72]. Among these potential targets is CD38. In a recent study, melanoma was induced in two different mouse models through the injection of tumor cells. The inhibition of CD38 was found to restrict the growth of primary tumors in both models, and the CD38-inhibited models also showed a lower rate of pulmonary and brain metastases. This was achieved through the promotion of cell death, reduction in cancer-associated fibroblast (CAF) numbers and the prevention of angiogenesis. CD38 is involved in angiogenesis via the production of NAADP [24]. In a previous study, melanoma-infected mice treated with an NAADP inhibitor called Ned-19 experienced inhibited melanoma growth, vascularization and metastasis, compared with untreated controls that developed well-vascularized tumors and pulmonary metastases [25,26]. This suggests that, at least in part, the effect of CD38 inhibition on melanoma is due to the reduction of NAADP production [24]. As CAFs and angiogenesis encourage tumor growth and metastasis [24,27], the anti-tumor effect of CD38 suppression can be explained by the removal of tumor-supporting elements in the TME. 

Another tumor-suppressive mechanism mediated by CD38 inhibition may be a reduction in TME adenosine levels. The sequential activity of CD38, CD203a, and CD73 results in the production of adenosine [18], and extracellular adenosine is known to be a powerful inhibitor of the anti-tumor immune response in melanoma. Primary melanoma cell lines have been found to suppress the proliferation of CD4+ and CD8+ T cells through an adenosine-dependent mechanism, but the use of CD38 and CD73 inhibitors have been found to reverse this effect, resulting in the restoration of T cell proliferation [35]. Thus, blocking the CD38-mediated adenosinergic pathway appears to reduce immunosuppression in melanoma [24].

The inhibition of CD38 appears to suppress melanoma progression through multiple mechanisms and pathways, making CD38 a potent therapeutic target for the treatment of melanoma. It may also represent a useful biomarker, as CD38 and HLA-DR are transiently coexpressed on EECs in melanoma [20]. This may allow clinicians to monitor the immune response against melanoma. 

#### 2.1.4. CD38 in Pancreatic Ductal Adenocarcinoma

Pancreatic ductal adenocarcinoma (PDAC) is one of the most aggressive forms of cancer, and has a particularly low 5-year survival rate of just 8% [48,73]. Due to its lack of early stage clinical manifestations, the majority of patients are diagnosed with PDAC when the disease has already reached advanced, metastatic stages. Surgical resection, the only potentially curative form of treatment, does not lead to any substantial improvement in patient prognosis at these stages of the disease [48]. Treatment options are thus limited to cytotoxic chemotherapy, which only improves life expectancy by a few months and results in severe side effects [73]. Furthermore, compared with other types of cancer, PDAC does not respond well to conventional treatment modalities, such as chemotherapy and targeted therapies due to the development of treatment resistance [74]. Thus, there remains a pressing need to develop novel treatment methods that can improve the survival rate of patients with PDAC.

Immunotherapy has shown significant therapeutic potential in other forms of solid cancers, and research is currently being conducted to investigate its potential for application in PDAC. However, trials using checkpoint inhibitors to treat PDAC, such as anti-CTLA-4 and anti-PD-1 therapies, have only elicited slight improvements in patient outcome [48,73]. A previous study has revealed that CD38 and CD101 are significantly upregulated on PD-1+ peripheral cytotoxic T cells in patients with PDAC compared with healthy donors, and higher levels of CD38 and CD101 coexpression on TILs were observed in the terminal stages of PDAC compared with the earlier stages. This coexpression could indicate the exhaustion of TILs, and it is correlated with decreased survival rates across all stages of PDAC [48]. In addition, late-stage PDAC tumors have increased numbers of CD38+/CD101+ infiltrates compared with those in the earlier stages [48]. As such, CD38 and CD101 coexpression on TILs may be a useful marker indicating adverse prognosis in PDAC.

#### 2.1.5. CD38 in Glioma

Gliomas are tumors of glial cells in the central nervous system (CNS). They are the most common primary tumors of the brain, accounting for 80% of all malignant brain tumors [51,52,75]. Glioblastoma, an aggressive form of glioma, is characterized by widespread invasion throughout the brain tissue, rendering surgical resection futile. Glioblastomas also show resistance to both conventional and more recently developed targeted treatments, such as cytotoxic chemotherapy. The outlook for glioblastoma patients is thus grim, with most surviving for up to a year with surgical and conventional treatments [52,76]. Novel treatment methods are urgently required for glioma patients, and immunotherapy targeting elements of the glioma TME may represent a potential solution. 

One of the reasons why gliomas are so difficult to treat is that they develop in the inaccessible, highly specific environment of the CNS. The glioma TME is known to be essential for promoting tumor growth and development [51,52]. Tumor-associated microglia/macrophages (TMMs) make up 40% of the tumor mass in gliomas [51,77], and are the most commonly occurring cells in the glioma TME [52]. A small proportion of these TMMs originate from resident brain microglia, while the majority come from infiltrating monocytes that become TMMs. TMM number is positively correlated with glioma grade and invasiveness [51,52]. Hence, TMM in the glioma TME may represent a useful prognostic tool.

CD38 has been discovered to regulate TMM activation through cADPR-mediated increase in calcium concentration [49,50]. Microglia in the CNS express CD38, and this expression is increased in glioma [50]. There is substantial proof to suggest that TMMs contribute to the immunosuppressive TME, and so promote the growth and metastasis of glioma. Matrix metalloproteases (MMPs) and other extracellular matrix-degrading enzymes released by TMMs support tumor proliferation. In addition, cytokines secreted by TMMs, such as interleukin-1, basic fibroblast growth factor, and VEGF, promote tumor progression and angiogenesis [52,53,54]. This suggests that inhibiting the pro-tumor activity of TMMs may be a promising approach for the treatment of glioma. This could potentially be achieved by targeting CD38.

Previous studies where glioma cells were injected into CD38-deficient mice revealed that the absence of CD38 in the TME brought about a reduction in tumor size and an improvement in life expectancy [51,52]. These positive effects were associated with an increased rate of tumor cell death and altered TMM activity. The production of MMPs by TMMs in these experiments was found to be reduced. Therefore, it seems that CD38 inhibition impedes glioma growth by suppressing the pro-tumoral activity of activated TMMs. These findings suggest that inhibition of CD38 should be explored as a potential therapeutic approach to treat glioma.

#### 2.1.6. CD38 in Breast Cancer

Breast cancer is the second most common cancer globally [78], and although much progress has been made in terms of treatment, the heterogeneity of certain types of breast cancer poses as a significant barrier to effective treatment [55,79,80]. Triple negative breast cancer (TNBC) is a form of breast cancer that does not express estrogen and progesterone receptors, or human epidermal growth factor receptor 2, all of which are widely-used therapeutic targets [81]. TNBC makes up just 10–15% of breast cancer cases [55], but is associated with poor prognosis and survival due to its aggressive nature and lack of molecular targets [82,83]. Thus, novel factors involved in the development and progression of TNBC must be identified, as well as those that can be used as markers for cancer prognosis and therapy.

In addition to the aforementioned types of cancer, CD38 may represent a potential prognostic factor in TNBC. Plasma cells can be identified by CD38 expression, and a previous study has demonstrated that higher CD38+ plasma cell density within TNBC tumors is correlated with higher disease-free survival (DFS) and overall survival rates. Every 1% increase in intratumoral CD38+ plasma cell density is associated with increased DFS [55]. CD38 may thus be a useful prognostic marker in TNBC. 

### 2.2. CD157 in the Solid Tumor Microenvironment

#### 2.2.1. CD157 in Ovarian Cancer

Ovarian cancer is the most lethal gynecologic malignancy, and is responsible for 4% of all cancer-associated mortality in women [84,85]. Epithelial ovarian cancer is a subgroup of ovarian cancer that arises from the surface epithelial lining of the ovaries and postovulatory inclusion cysts [37,40], and accounts for >85% of ovarian cancer cases [84,86]. The relatively poor prognosis of this condition is primarily due to its asymptomatic presentation in the early stages, with symptoms normally emerging only after the tumor becomes widely metastatic in the abdomen [37,40,87]. Treatment for ovarian cancer usually involves both surgery and chemotherapy; however, even with multiple rounds of treatment relapse is almost inevitable [84,85]. The majority of patients with advanced epithelial ovarian cancer experience relapse within 1.5 years after treatment [87].

As such, there is a need for better prognostic tools and more effective therapeutic approaches against this type of cancer. CD157 plays a key role in ovarian cancer invasiveness and metastasis through its expression on ovarian cancer cells, where it regulates the interaction of these cells with the mesothelium and extracellular matrix proteins [37]. CD157 is known to control leukocyte adhesion, migration and diapedesis [10,37] and multiple previous studies suggest that CD157 expression on ovarian cancer epithelial cells allows for the spread and movement of tumor cells, in the same way that it enables leukocyte trafficking. For example, experiments comparing CD157+ and CD157- ovarian cancer cells showed that the loss of CD157 expression on tumor cells inhibited tissue invasion and severely limited tumor cell migration [37,40]. The use of mAbs targeting CD157 also results in reduced tumor cell adhesion to proteins in the extracellular matrix, including type 1 collagen, fibronectin, and laminin [40]. Analysis was also done to investigate changes in gene expression following CD157 transfection in ovarian cancer cells, which were found to acquire mesenchymal traits. This indicates that high expression of CD157 strengthens processes that promote tumor cell proliferation and migration. Furthermore, processes that inhibit tumor growth, such as apoptosis, were reduced [56]. Increased expression of CD157 on ovarian cancer cells is also correlated with increased malignancy and greater risk of relapse [37,40].

Thus, the results of these previous experiments consistently show that CD157 directly implicated that it may represent a useful prognostic tool and a target for the treatment of ovarian cancer.

#### 2.2.2. CD157 in Pleural Mesothelioma

Malignant pleural mesothelioma (MPM) is an aggressive type of cancer that arises from the lung pleura. The most common cause of MPM is exposure to asbestos. Despite it being a rare disease, the number of MPM cases has increased significantly over the past few decades due to the unregulated use of asbestos in developing and industrial countries [88,89,90]. Many patients with MPM are diagnosed at a late stage, after pleural dissemination has taken place, which results in a relatively poor prognosis. The median survival of MPM patients is 12 months, and complete remission is rare [57,91]. The therapeutic approaches currently used for MPM include chemotherapy, surgery and radiation therapy, but none of these are curative and yield only limited increases in life expectancy [89,90,91]. As such, novel forms of treatment for MPM are currently being investigated, with immunotherapy showing particular promise [89,90].

As aforementioned, CD157 is a glycoprotein involved in ovarian cancer invasiveness and metastasis [56]. Since ovarian epithelial cells and mesothelial cells share a common embryonic origin, this suggests that CD157 may also serve a role in MPM progression. Furthermore, CD157 expression was observed in >85% of MPM tissue samples investigated by a previous study [57]. The presence of CD157 has also been found to be associated with increased malignancy in biphasic MPM. CD157+ and CD157-knockdown cell lines were compared, and CD157 expression on biphasic MPM cell lines was found to be associated with increased cell proliferation, invasiveness, and dissemination, as well as a higher level of resistance to platinum-based chemotherapy. Biphasic MPM tumors have both epithelial and mesenchymal constituents, suggesting the occurrence of an epithelial-to-mesenchymal transition (EMT) process [57]. EMT is associated with increased malignancy in cancer, as it confers greater invasiveness and motility to tumor cells and facilitates metastasis [92]. Extracellular matrix proteins such as fibronectin promote EMT [93], and since CD157 mediates adhesion to these proteins [12], expression of this glycoprotein increase cancer malignancy by promoting mesenchymal differentiation [56]. A higher level of CD157 expression is therefore an indication of poor prognosis in patients with biphasic MPM.

CD157 may also be involved in the activation of the mTOR pathway in MPM. The mTOR pathway promotes tumor progress by increasing cell proliferation and protein synthesis [94], and this leads to resistance to chemotherapeutic drugs [94]. An association has been found between the phosphorylation of mTOR and the presence of CD157 in biphasic MPM, suggesting that CD157 expression may be associated with mTOR pathway activation [57]. Due to this association with both EMT and mTOR pathway activation, the presence of CD157 may be used to stratify MPM patients into different prognostic groups, and to select the most appropriate therapeutic approaches for different patients.

#### 2.2.3. Potential Functions of CD157 in Other Types of Tumor

The role of CD157 in ADPR production suggests that it may be involved in the CD38/CD157/CD203a/CD73 adenosinergic pathway [35]. The production of extracellular adenosine results in an immunosuppressive TME and thus favors tumor progression [18,19]. This may be of relevance in cancers such as HCC, NSCLC, and melanomas, in which the production of adenosine has been shown to promote tumor progression through immunosuppression. Furthermore, CD157 has been found to be expressed in the TME of HCC (Figure 2). More research has to be done to confirm the adenosinergic role of CD157 in these cancers, but it may also represent a promising target for immunotherapy for these diseases in the future.

## 3. Conclusions

The progression and aggressiveness of a cancer depends on not just the nature of the tumor cells themselves, but also the TME. The TME consists of extracellular matrix proteins, immune cells, vascular cells, lymphatics and fibroblasts, and is of great importance to a cancer’s responsiveness to immunotherapy. CD38 and CD157 glycoproteins, which are expressed on tumor cells and immune infiltrates, serve a pertinent role in the promotion of tumor progression across multiple kinds of cancer, but may also represent a therapeutic solution. 

CD38 has long been considered an immune molecule due to its ubiquitous expression across the immune system, and previous studies have shown that CD38 serves a complex, multifactorial role in the promotion of tumor growth and resistance to cancer immunotherapy. CD38 is known to be expressed on immune infiltrates in the TME of multiple types of solid tumor, where it exerts an immunosuppressive effect through multiple mechanisms. CD38 also mediates resistance to certain forms of cancer immunotherapy. This review evaluates and integrates current data on the roles of CD38 in several cancer types including HCC, NSCLC, melanoma, pancreatic cancer, glioma and breast cancer. Based on this data, this review concludes that the role of CD38 can be both pro-tumoral and anti-tumoral. This is highly dependent on the cancer type, cell type, and interplay among various cell types that are present in the microenvironment. Further studies are required to explore and uncover the complexities and interplay of these factors.

On the other hand, CD157, which has similar functions and properties, has been found on ovarian and MPM tumor cells. Its presence on these tumor cells is consistently associated with increased tumor invasiveness and metastasis. 

The above findings suggest that both CD38 and CD157 represent promising prognostic tools and intriguing targets for immunotherapy in solid tumors, and are worthy of further investigation in this regard.

## Figures and Tables

**Table 1:** Functions of CD38 in different types of cancer.

Tumor Type	Cell Type	Function of CD38
Hepatocellular carcinoma	Tumor infiltrating lymphocytes (B and T cells) 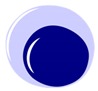	- Presence of CD38+ TILs within the TME increases effectiveness of cancer immunotherapy [2,7], as activated CD38+ TILs induce inflammation and promote an anti-tumor immune response [7] via the production of cytotoxic compounds and inflammatory cytokines [45,46]
	Tumor cell 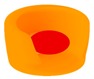	- CD38 upregulation on tumor cells suppresses cytotoxic T cell function due to its adenosinergic activity, thus limiting the therapeutic effect of anti-PD-1 immunotherapy [2,44]
	Macrophages 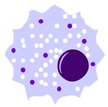	- CD38+ macrophages in the TME may promote inflammation and exert anti-tumor effects. This is because CD38 expression is associated with M1 macrophages, which produce pro-inflammatory cytokines [47] - Coexpression of CD38 and CD68 on macrophages is correlated with improved prognosis after surgical resection by increasing the M1 to M2 macrophage ratio [30]
Non-small cell lung cancer	Tumor cell 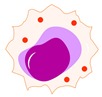	- CD38 upregulation on tumor cell correlates with development of resistance to immunotherapy. This is because CD38 suppresses activity of cytotoxic T cells via the adenosinergic pathway [44]
	T cell 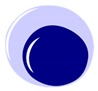	- CD38 is expressed by early effector T cells, hence it serves as a biomarker for the monitoring of the immune response against NSCLC [31]
Melanoma	Tumor microenvironment 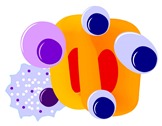	- CD38 is a potential immunotherapy target which can be inhibited to restrict growth of primary tumors [24] - CD38 inhibition prevents tumor angiogenesis and metastasis [25,26,27] - CD38 mediates suppression of cytotoxic T cell activity via the adenosinergic pathway [24,35]
Pancreatic ductal adenocarcinoma	Tumor infiltrating lymphocytes - B and T cells 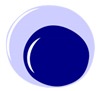	- CD38 and CD101 coexpression on TILs could indicate TIL exhaustion, and hence is a marker for adverse prognosis [48]
Glioma	Tumor-associated microglia/macrophages (TMM) 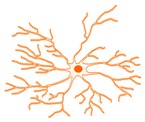	- Regulates TMM activation through cADPR-mediated increase in calcium concentration [49,50], hence serves as a prognostic tool as TMM number is positively correlated with glioma grade and invasiveness [51,52] - Creates an immunosuppressive tumor microenvironment via TMM activation, as TMM promote tumour angiogenesis and metastasis through the secretion of cytokines [52,53,54]
Breast cancer	Plasma cell 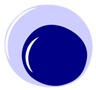	- High CD38 expression and high CD38+ plasma cell density are correlated with increased rates of disease free-survival and overall survival [55], and hence CD38 plays a prognostic role in triple negative breast cancer

**Table 2:** Functions of CD157 in different types of cancer.

Tumor Type	Cell Type	Function of CD157
Ovarian cancer	Epithelial 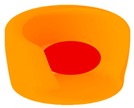	- Promotes tumor cell invasion and migration [37,40], by reducing tumor cell binding to extracellular matrix proteins [40] - Correlated with increased malignancy and greater risk of relapse [37,40], as it promotes tumor cell acquisition of mesenchymal traits and reduces apoptosis [56]
Pleural mesothelioma	Mesothelial 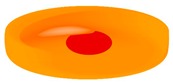	- Increases tumor cell proliferation, invasiveness, and dissemination by activating the mTOR pathway [57] - Promotes mesenchymal differentiation [56] by mediating adhesion to extracellular matrix proteins [12]
